# Effect of On-Pump Versus Off-Pump Surgery on the Renal Function of Diabetic Patients: A Systematic Review

**DOI:** 10.7759/cureus.93308

**Published:** 2025-09-26

**Authors:** Aminahmad Bolourchi, James Bosire Sagini Oroko, Mohamed Hussein, Anastasia Solomou, James Tang

**Affiliations:** 1 Medicine, Royal Free Hospital, London, GBR; 2 Medicine/Oncology, Royal Free Hospital, London, GBR; 3 Cardiology, Royal Free Hospital, London, GBR

**Keywords:** cardiothoracic surgery, coronary artery bypass grafting (cabg), diabetes mellitus, off-pump cabg, on-pump cabg, renal function, systematic review

## Abstract

The effects of on-pump versus off-pump cardiac surgery on postoperative renal outcomes of diabetic patients remain unclear. Using prospective and retrospective clinical studies, we explored the impact of diabetic nephropathy (DN) on renal outcomes in this patient population. Additionally, we considered the impact of renal insufficiencies caused by DN on the renal outcome.

We searched electronic databases using predetermined keyword combinations. A total of 8,083 studies were identified, of which 11 met the prespecified inclusion and exclusion criteria. The remaining nine observational studies and two randomised controlled trials were examined and extrapolated in the review matrix for critical analysis.

The literature search generated 11 relevant studies, and the preoperative parameters were compared between the on-pump and off-pump groups in the studies to establish the influence of confounding variables. Statistically insignificant trends were identified, indicating a reduced incidence of renal failure and renal dysfunction in diabetic patients who underwent off-pump surgery. There was no difference in the outcome of severe renal failure requiring renal replacement therapy between the different surgical strategies. A trend favouring off-pump surgery was extrapolated in diabetic patients with preoperative renal insufficiency.

We concluded that off-pump coronary artery bypass grafting (CABG) offers comparable long-term outcomes to on-pump CABG while demonstrating potential advantages in reducing perioperative complications, particularly in high-risk patients. However, further large-scale randomised trials are warranted to confirm these findings and guide individualised surgical decision-making.

## Introduction and background

This systematic review focuses on diabetic patients, aiming to compare the effect of off-pump surgery as opposed to cardiopulmonary bypass (CPB) on their renal function, evaluating the impact of pre-existing diabetic nephropathy (DN) on the renal outcomes of coronary artery bypass grafting (CABG) surgery. The review compares the evidence on the renal outcomes of diabetic patients who underwent either on-pump coronary artery bypass graft (ONCABG) or off-pump coronary artery bypass graft (OPCABG) surgery and analyses the data against the null hypothesis, "there is no difference in the postoperative renal outcome of diabetics who undergo ONCABG or OPCABG".

Cardiopulmonary bypass

CPB is a surgical technique used to provide a bloodless field for cardiac surgery. CPB components and conduct are optimised for myocardial, renal, cerebral, and pulmonary protection. Successful conduct requires a well-coordinated joint team effort of the surgeon, perfusionist, and anaesthesiologist.

**Figure 1:**
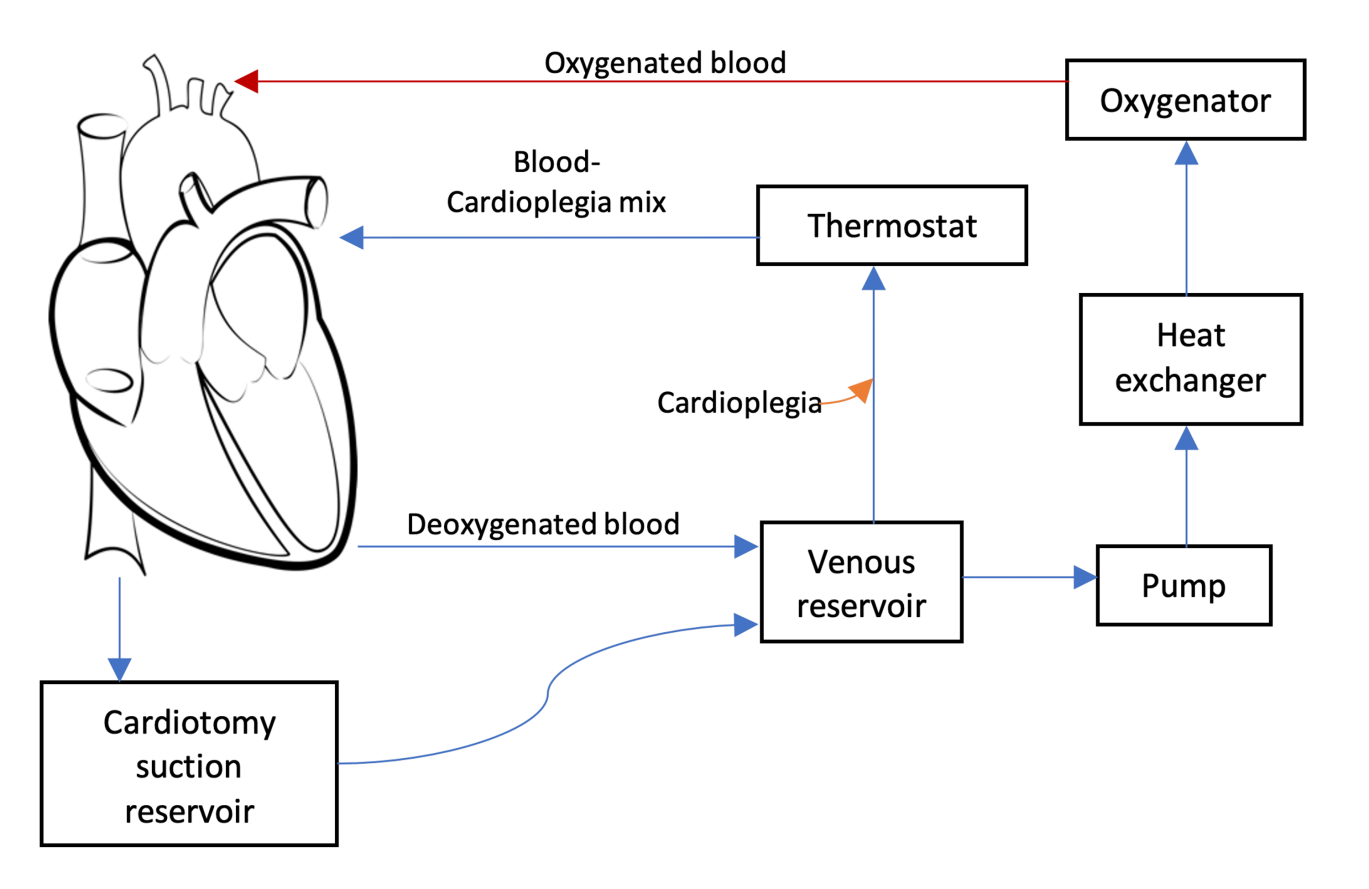
Schematic diagram highlighting the main components of the cardiopulmonary bypass circuit that are discussed in this review. Image created by the author.

The CPB circuit comprises a number of main components that have been highlighted in Figure 1, and each is specialised to carry out specific functions. The pump is responsible for creating the flow of blood. The roller pumps use two rollers positioned on a rotating arm to pump blood. Alternatively, centrifugal pumps use impellers to create rotational energy that pressurises the tubing, causing unidirectional flow. These pumps create non-pulsating flow by exerting force on the CPB circuit tubing, creating a pressure gradient, which can damage erythrocytes. Whilst the centrifugal pump is less destructive to blood components, both pumps cause haemolysis to a certain extent [1]. The type of CPB pump used may also impact the haematocrit, as clinical comparisons have shown a lower mean priming volume associated with roller pumps, causing less haemodilution [2]. The circuit tubes are commonly made of polyvinyl chloride (PVC) and are used to maintain circulating blood, risking immune activation by the ‘foreign’ surface interaction. Membrane oxygenators are the most commercially used form of oxygenators in CPB. Diffusion of blood and gases at the hollow microporous polypropylene fibres of this device enables effective exchange of gases and blood oxygenation. These hollow fibres filter the escape of air into the circuit, reducing the risk of air embolisms [1]. The reservoir's main function is to hold venous blood in the circuit. These can either be an open cardiotomy suction reservoir or closed reservoirs that require an additional circuit to process suctioned blood. Open reservoirs have the additional advantage of passively removing venous entrapped air, but closed reservoirs provide a smaller surface area in contact with blood components, which reduces inflammatory activation. The heat exchange controls the rate of heat exchange in the blood running through the circuit, maintaining optimal serum temperature for the surgical procedure.

In the initiation of CPB, heparin is administered intravenously to adjust the activated clotting time (ACT) to the target range of >480 seconds, reducing the risk of thromboembolism. This is followed by aortic cannulation, cross-clamping, discontinuation of ventilation, and the start of cross-clamp time. ACT should be monitored continually during CPB, as well should the perfusion pressure, to avoid blood coagulation and ischaemia, respectively. Hypoperfusion of the kidneys can precipitate acute kidney injury (AKI) through ischaemia/reperfusion (I/R) injury. Despite the compensatory effects of haemodilution on increased flow resistance, hypothermic CPB conditions naturally increase blood viscosity, which means that the perfusion pressure must be maintained at its upper limit [3]. Additionally, during CPB, haemofilters are used to remove inflammatory mediators and excess blood fluids. Removal of inflammatory mediators can reduce postoperative AKI by minimising the CPB-mediated systemic inflammatory response syndrome.

Off-pump surgery

Beating heart surgery demands highly precise and delicate hand-work to sew on high-quality anastomoses, making the operation more complex than on the cardioplegically arrested heart. Consequently, off-pump surgery is only utilised in CABG cases where patients are at higher risk of CPB complications or aortic manipulation [4]. Off-pump surgery eliminates the need for extracorporeal circulation, an advantage that directly correlates with reduced pre- and postoperative disruption of blood glucose homeostasis [5,6]. The lower levels of hyperglycaemia in off-pump diabetic patients require less rigorous control, making the procedure more feasible in terms of management. Moreover, hyperglycaemia is a driving factor for diabetic microvascular diseases, such as renal nephropathy, that cause postoperative renal dysfunction.

The pulsatile flow created by the heart makes off-pump surgery ideal for preserving kidney function in patients suffering from pre-surgical renal dysfunction [7]. These and other factors are further explored in this review to better understand the impact of these techniques on diabetic patients who are at higher risk of kidney disease due to the renal implications of persistent hyperglycaemia [8].

Diabetes and diabetic nephropathy

According to the World Health Organisation, 8.5% of the global population had diabetes mellitus in 2014, and this figure has been climbing ever since [9]. Diabetes can directly precipitate kidney dysfunction in patients. An estimate of 20% to 40% of diabetic patients go on to develop DN in their lifetime [10]. Translating these data to surgical practice, it can be assumed that many individuals undergoing CABG suffer from diabetes with potential underlying DN.

**Figure 2:**
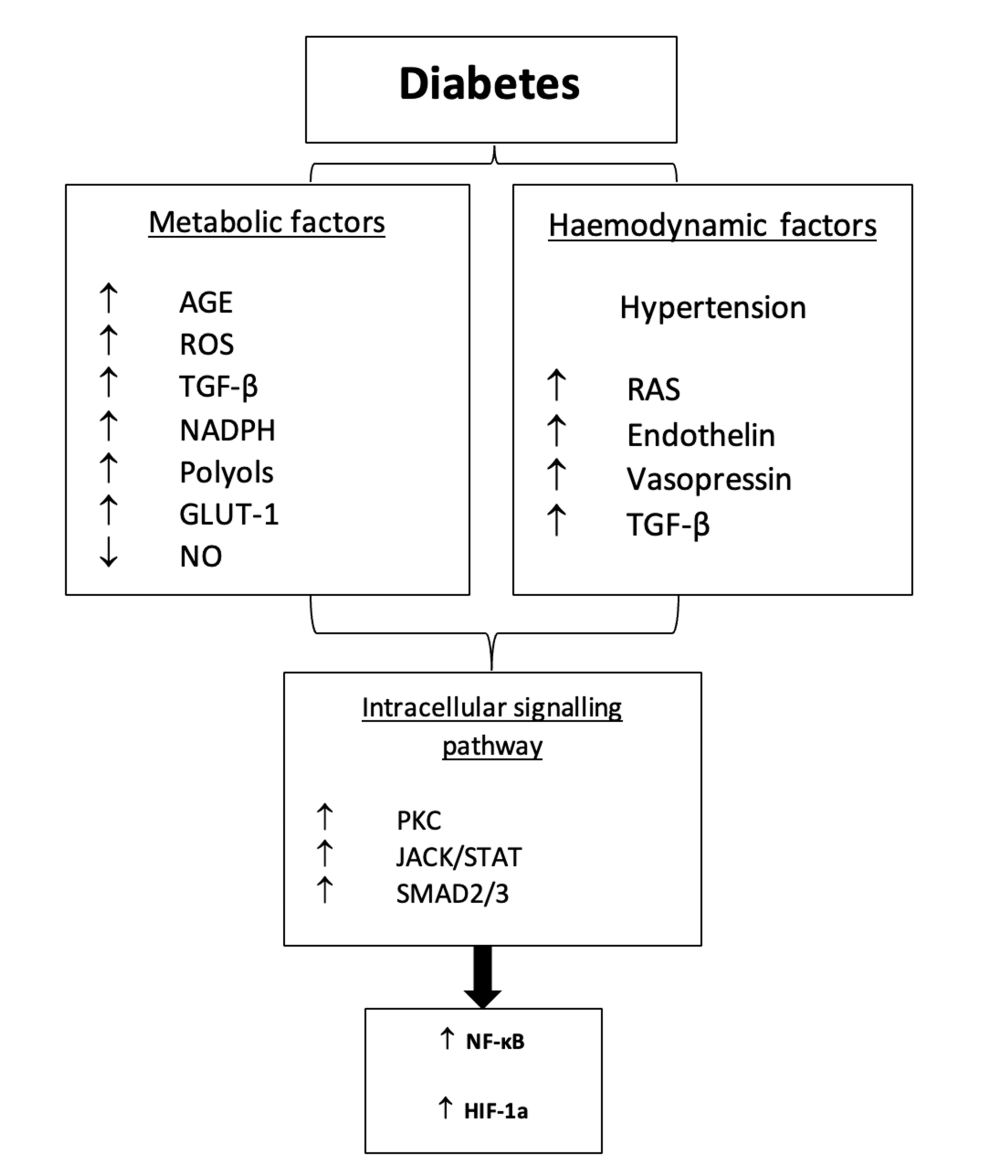
Different pathways involved in diabetic nephropathy disease initiation and progression, influencing the activation of transcription factors involved in inflammatory pathways. AGE, advanced glycation end products; ROS, reactive oxygen species; TGF-β, transforming growth factor-beta; NO, nitric oxide; RAS, renin angiotensin system; PKC, protein kinase C; NADPH, nicotinamide adenine dinucleotide phosphate; NF-κB, nuclear factor-κB. Image created by the author using resources [11-14].

Diabetes is a metabolic disorder that drives chronic hyperglycaemia through two different modes of disease manifestation. Insulin-dependent diabetes mellitus (IDDM; type 1) is caused by impaired insulin production by the beta cells located in the islets of Langerhans of the pancreas. Non-insulin-dependent diabetes mellitus (NIDDM; type 2) progressively develops due to an imbalance between insulin secretion and insulin sensitivity within the body. DN is a condition characterised by albuminuria, reduced glomerular filtration rate (GFR) and formation of glomerular lesions. As indicated by Figure 2, DN pathophysiology is a consequence of multiple diabetic metabolic and haemodynamic factors that damage endothelial cells of the renal circulation, podocytes, and tubular cells through activation of inflammatory pathways.

Endothelial dysfunction is a major contributor to DN pathology. In DN, endothelial nitric oxide (NO) synthase uncoupling and elevation of proximal reactive oxygen species (ROS) reduce the NO production, which in turn renders the vasculature more sensitive to vasoconstriction by mediators such as endothelin-1 [11,12]. The increased oxidative stress on endothelial cells triggers nuclear factor-κB (NF-κB) signalling, which promotes apoptosis, reducing vascular density (known as vascular rarefaction) [13]. A combination of this outcome and vasoconstriction creates a hypoxic environment that is specifically damaging to the proximal tubular cells due to high metabolic demand. Hypoxia promotes mitochondrial dysfunction and the activation of HIF-1ɑ transcription factor. HIF-1ɑ contributes to fibrogenesis and facilitates endothelial to mesenchymal transition in DN [14]. The hypoxic and pro-inflammatory environment created in the kidney further promotes ROS production and renin-angiotensin system (RAS) activation. These factors drive podocyte apoptosis and disrupt the glomerular filtration barrier, enabling larger proteins to pass through the Bowman’s capsule, precipitating albuminuria. Activation of NF-κB can also mediate glomerulosclerosis, mesangial expansion, and glomerular hypertrophy, which are all histologic features of DN.

Diabetic patients undergoing surgery are at higher risk of AKI due to factors driven by DN, which will be further explored in this review. AKI causes kidney fibrosis due to pathological and maladaptive repair mechanisms of DN, and repetitive exposure to AKI can eventually accumulate to cause end-stage renal disease (ESRD).

## Review

Materials and methods

The Critical Appraisal Skills Programme (CASP©) Appraisal tool was consulted to ensure that this systematic review met the ‘CASP Systematic Review Checklist’ requirements. Organisation of the systematic analysis was achieved to enhance accessibility by compartmentalisation of extracted data into folders such as ‘PRISMA’, ‘Review Matrix’, ‘Data collection’, ‘Project theory’, and ‘Tables and figures’.

The literature search commenced immediately after the establishment of the research hypothesis and targets. Results were filtered to all studies from the year 2000 to June 2025. The studies considered in this review were collected via an internet literature search using the following keywords: ‘Off-pump’, ‘On-pump’, ‘Surgery’, ‘Diabetic’, ‘Renal’, and ‘Outcome’. The search terms were used in different combinations to generate 8,083 results across Medline (PubMed), ScienceDirect, ClinicalTrials, the National Institute for Health and Care Excellence (NICE), and Cochrane Library. Additionally, three studies were identified through ‘snowballing’, a process where the reference list of studies is searched to identify novel studies.

**Figure 3:**
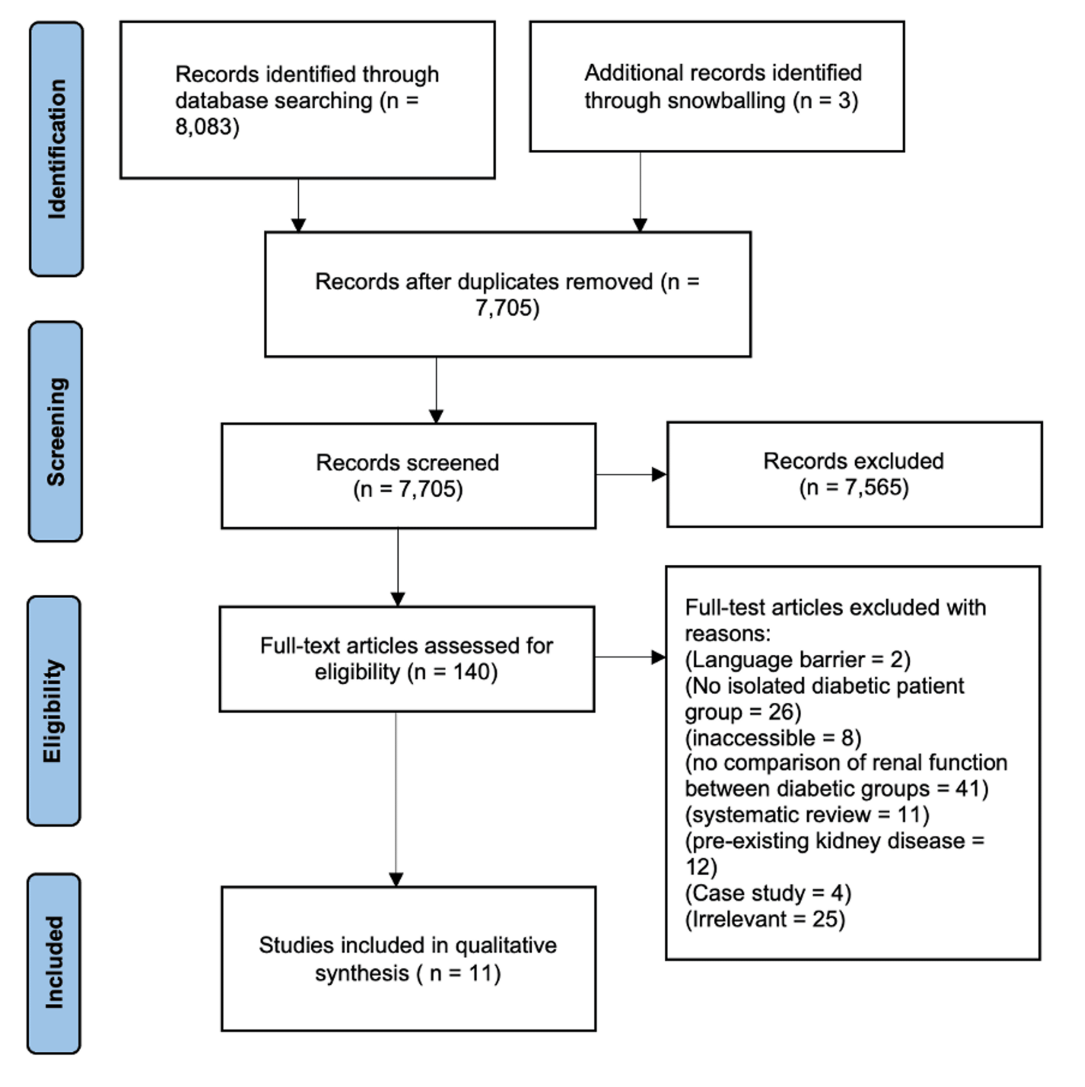
PRISMA flowchart depicting the flow of articles from the initial identification stage to the inclusion of contributing studies. PRISMA, Preferred Reporting Items for Systematic Reviews and Meta-Analyses.

As indicated in Figure 3, during the first step of screening, 378 duplicate entries were identified and discarded by cross-checking title and author information across databases. The next step was the screening of entries by title relevance, which further eliminated 7,565 studies that did not contribute to the research question. Finally, the remaining 140 studies were analysed by abstract against the pre-determined study inclusion/exclusion criteria. A total of 129 studies were removed, leaving 11 valid studies to be used in this systematic review.

Inclusion/Exclusion Criteria

The inclusion/exclusion criteria were decided during the research theory period of the study, presented in Table 1. These highlight the key variables that must exist in a relevant study, and the variables that, if present, impair the efficacy of this review. All the included studies compare on-pump and off-pump surgery in diabetic patients with respect to renal function, account for diverse preoperative risk factors (including preoperative renal function, gender, and age), and apply appropriate statistical tests to interpret the data. Case reports and retrospective cohort studies, due to their retrospective nature, were excluded from this review. Their inclusion would have restricted the investigator from controlling preoperative patient characteristics and increased the risk of publication and selection bias that would have negatively influenced the cause-and-effect relationship. The comparison of the renal outcome of renal insufficient patients undergoing the different cardiac surgery strategies was made independent of the literature search.

**Table 1:** Inclusion/exclusion criteria used to screen studies and check for their eligibility.

Inclusion	Exclusion
Published data between 2000 and 2025	Literature review
Primary research	Case report
Prospective review	Studies without statistical tests on diabetic patient groups
Study group of diabetic patients (type 1 or type 2) or with a subgroup of diabetic patients (type 1 or type 2)	Meta-analysis
Data gathered on both on-pump and off-pump strategies	Comment/letter/correspondence to the editor
English language as the original; alternatively, a translated version is available	Single gender study
Outcome measured is renal failure, chronic kidney disease, acute renal failure, or renal replacement therapy	Study conducted on diabetic patients with pre-existing end-stage renal disease
Retrospective analysis of prospective data	No pre-surgical kidney assessment

The included studies consisted of primary studies, randomised clinical trials, and cohort studies published between 2000 and 2018. A thorough analysis of these studies was conducted and summarised in the Review Matrix Appendix, which is submitted as a supplementary file in the Appendix. The appendix aims to highlight the vital and recurring themes within the included studies, with the incorporation of appropriate CASP checklist questions, emphasising study design, limitations, strengths, outcomes and statistical work.

Results

A total of 10 studies were extracted following the literature search and included in the analysis. All of these studies partially or entirely focused on diabetic patients, accounted for relevant preoperative parameters that could confound outcomes, and compared the difference in postoperative renal function in patients who underwent ONCABG or OPCABG surgery. The main preoperative parameters considered to influence postoperative AKI were gender, age, renal dysfunction, body mass index (BMI), and the number of diseased vessels to treat (reasoning for inclusion is presented in *Confounding Factors*). The studies included measured outcomes denoting the status of renal function, such as renal failure (RF) with or without the need for dialysis, renal replacement therapy (RRT), haemofiltration, creatinine clearance (CrCl), and proteinuria. This section will focus on systematic and quantitative assessment of the different measures of renal function after surgery, with consideration of the different pre-surgical characteristics of enrolled participants.

Outcomes of Renal Failure

**Figure 4:**
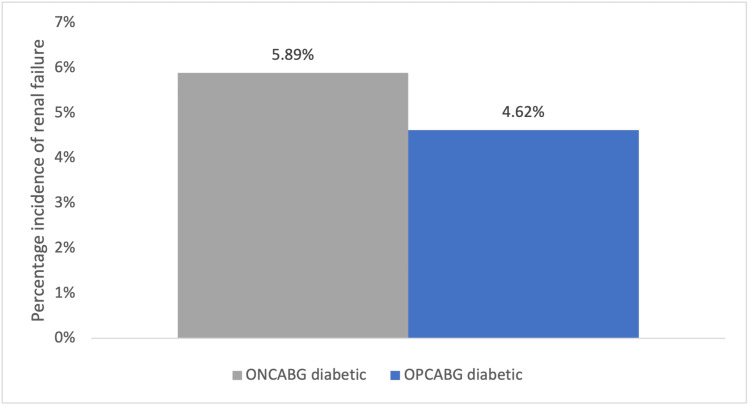
Percentage incidence of renal failure observed in diabetic and non-diabetic patients having undergone OPCABG or ONCABG surgery. OPCABG, off-pump coronary artery bypass graft; ONCABG, on-pump coronary artery bypass graft. Data from Magee et al. [15].

An observational study by Magee et al. measured the outcome of RF requiring dialysis (RF-dialysis) separate from RF alone, providing statistical comparisons within 2,891 diabetic patients and 7,074 nondiabetic patients. The preoperative parameters measured in the diabetic cohort showed statistically significant differences in mean age, greater in the OPCABG group (P = 0.0015); gender, 5.93% more females in the OPCABG group (P = 0.026); RF, 3.49% greater incidence in the OPCABG group (P = 0.0134); and obesity, 11.39% greater frequency in the ONCABG group (P = 0.0001) between the groups. The extent of vessel disease was not included as a parameter. The same pattern of preoperative patient characteristics found in the diabetic cohort was present in the nondiabetic cohort. No statistical adjustments were made to account for the difference in preoperative covariates. In the diabetic and nondiabetic cohorts, the presence of CPB did not impact the frequency of postoperative RF. Despite a 1.27% greater incidence of RF in the diabetic ONCABG group, no statistically significant difference between surgical strategies was observed (P = 0.3401). The overall comparison in Figure 4 shows a trend of higher RF incidence in diabetic patients undergoing ONCABG [15].

The third observational study in this review was conducted by Srinivasan et al. on 951 diabetic patients who underwent ONCABG or OPCABG [16]. To account for any surgical strategy conversion, the ‘intention-to-treat’ methodology was utilised. This method overlooks any changes that occur after the allocation of a patient to a group and will keep their data in the same group, despite any changes. The preoperative parameters measured in each group showed statistically significant differences in obesity, 1.1 kg/m^2^ greater BMI in the OPCABG group (P = 0.032), and three-vessel disease, 6.1% greater frequency in the ONCABG group (P = 0.036). No statistically significant differences were detected in patient age, gender, and renal dysfunction. Preoperative creatinine levels were not quantified between the groups. During the study period, two (1.075%) cases of OPCABG were converted to ONCABG. The statistical analysis of the crude postoperative data showed an RF incidence of 6.0% in ONCABG and 3.2% in OPCABG, without any statistical significance (P = 0.13). To cancel out the confounding impact of preoperative patient characteristics on postoperative data, propensity scores were generated to account for the covariates. The propensity score-adjusted postoperative results showed a statistically significant (P = 0.036) increase in the incidence of RF in ONCABG, with 6.3% compared to 2.7% in OPCABG [16].

An observational cohort study conducted by Emmert et al. retrospectively analysed the data derived from 1,015 diabetic patients who underwent OPCABG or ONCABG surgical strategy [17]. Overall, 52 preoperative patient parameters were quantified, which did not include CrCl. Patient groups showed a statistically significant difference in the preoperative number of diseased vessels, with 11.7% higher incidence of three-vessel disease in ONCABG (P = 0.001). No statistically significant difference in renal dysfunction, comorbidity, age, gender, and obesity was identified between the ONCABG and OPCABG surgical groups. The OPCABG group experienced a 5.6% treatment conversion to ONCABG, and to account for this variable, the study progressed with the intention-to-treat methodology. Postoperative crude data analysis showed a 2.2% lower frequency of RF in the OPCABG group, with the data showing a trend towards a reduction in postoperative RF (P = 0.10). Computation of 52 patient covariates in an adjusted regression model gave rise to the interpretation of data using propensity score adjustments. Propensity-adjusted RF outcome showed less of a trend in favour of OPCABG (OR (95% CI) = 0.57 (0.25-1.27); P = 0.17) [17].

**Figure 5:**
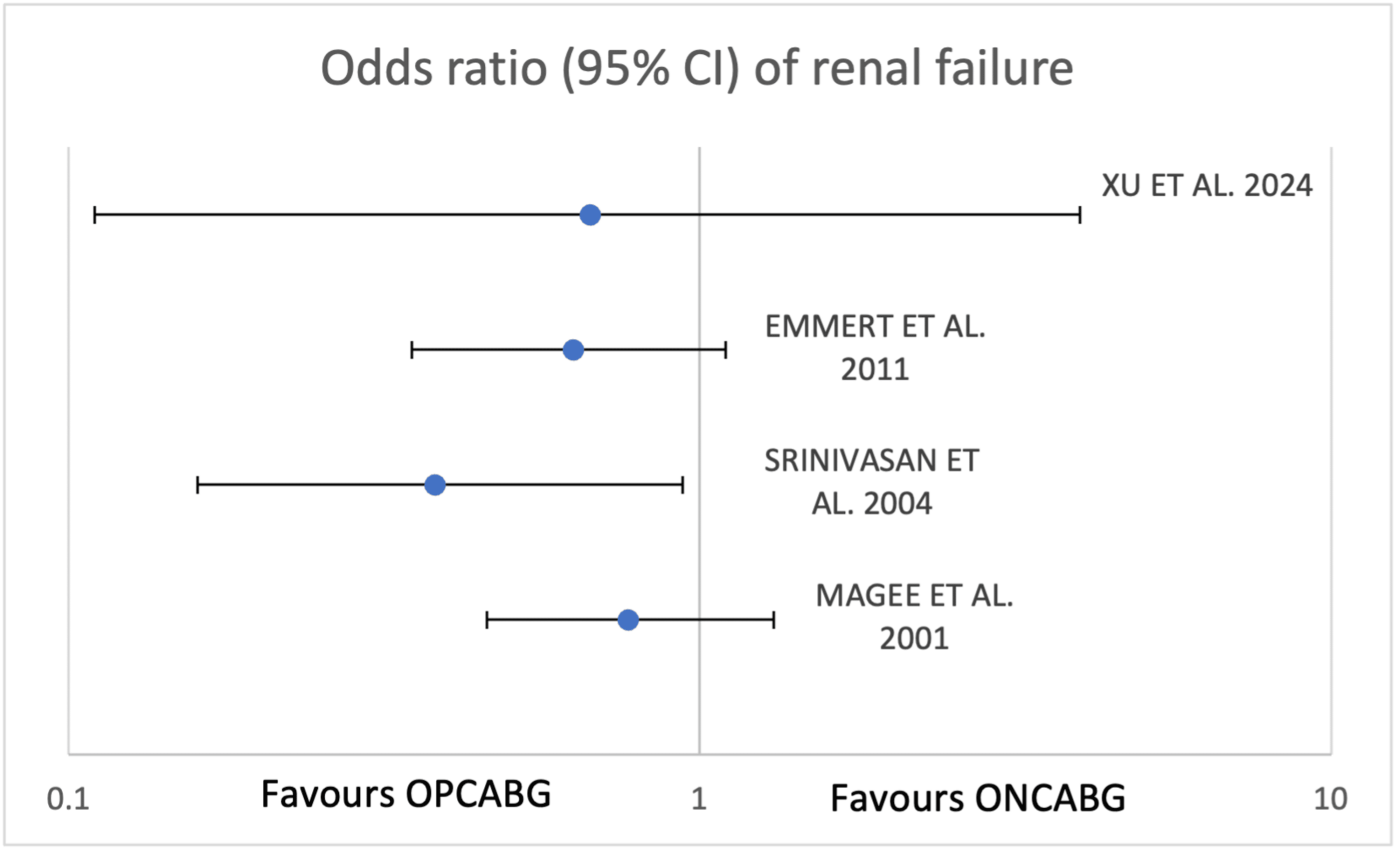
Forest plot of odds ratios of renal failure in diabetic patients. OPCABG, off-pump coronary artery bypass graft; ONCABG, on-pump coronary artery bypass graft. References [15-18].

A more recent contribution by Xu et al. (2024) further evaluated renal failure outcomes in diabetic patients undergoing OPCABG versus ONCABG through a large-scale retrospective study [18]. The analysis included 3,142 diabetic patients undergoing isolated CABG, with 1,485 receiving OPCABG and 1,657 receiving ONCABG [18]. After propensity score matching, 1,300 matched pairs were analysed to ensure balanced baseline characteristics. The incidence of postoperative renal failure was 3.5% in the ONCABG group and 2.7% in the OPCABG group, a difference that did not reach statistical significance (P = 0.224). Multivariable logistic regression confirmed that off-pump CABG was not independently associated with reduced risk of renal failure. These findings reinforce the consistency across multiple studies, suggesting that while OPCABG may yield subtle differences in renal outcomes, it does not provide a statistically significant advantage over ONCABG in preventing postoperative renal failure in diabetic patients (Table 2 and Figure 5).

**Table 2:** Studies comparing the event of postoperative renal failure against the total sample population undergoing different CABG treatments. Effect size and statistical comparison of outcome between groups presented as odds ratios (OR) and P-values, respectively. CABG, coronary artery bypass graft; OPCABG, off-pump coronary artery bypass graft; ONCABG, on-pump coronary artery bypass graft. Magee et al., Srinivasan et al., and Emmert et al. utilised a chi-squared model, whilst Xu et al. utilised a logistic regression model. Threshold of statistical significance of p < 0.05 [15-18].

	OPCABG		ONCABG			
Study	Events	Total	Events	Total	P-value	OR (95% CI)
Magee et al. (2001) [15]	16	346	150	2545	0.3401	0.77 (0.46, 1.31)
Srinivasan et al. (2004) [16]	5	186	48	765	0.036	0.38 (0.16, 0.94)
Emmert et al. (2011) [17]	22	540	30	475	0.1	0.63 (0.35, 1.10)
Xu et al. (2024) [18]	2	504	3	504	0.654	0.67 (0.11, 4.00)

Renal Replacement Therapy as a Measure of Severe Renal Failure

Post-surgical AKI can severely disrupt renal function, stimulating an urgent need for RRT in the intensive care setting. RRT includes the use of haemodialysis, haemofiltration, or haemodiafiltration. In principle, haemodialysis and haemofiltration are similar as both apparatuses use a semipermeable membrane to filter blood components, replacing the function of the kidneys.

An observational study conducted by Benedetto et al. analysed the short-term need for RRT in 2450 diabetic patients, of whom 48.9% underwent ONCABG and the rest OPCABG [19]. Comparisons of preoperative patient parameters between groups did not show any significant difference in age, gender, BMI and serum creatinine (SCr) >200 ml/L between the surgical groups. Patients undergoing ONCABG were associated with a higher frequency of three-vessel disease, but this variable was adjusted at baseline in the 995 propensity score-matched pairs. The postoperative propensity-adjusted outcomes of RRT were 4.9% in the OPCABG group compared to 4.3% in ONCABG (P = 0.52), indicating no significant difference between the two groups [19].

Shroyer et al.'s paper was the only multi-centred, prospective randomised controlled trial (ROOBY trial) included in this review. This study evaluated RF-dialysis as a secondary outcome in 835 male veteran diabetic patients who underwent CABG. The preoperative parameters measured between the OPCABG and ONCABG groups showed no statistical significance in coronary distribution of diseased vessels, age, SCr, and gender. There was no preoperative comparison of BMI between OPCABG and ONCABG. Perioperative conversion of OPCABG was 12.7% compared to 3.7% in ONCABG (P < 0.001), and the influence of surgical conversions was adjusted during the sensitivity analysis to ensure the findings were valid. The outcome of RF-dialysis between groups did not show any statistical significance (P = 0.903) with 1.15% and 1.25% frequencies in ONCABG and OPCABG, respectively. As presented in Table 3, Magee et al. reached a contradictory conclusion by presenting the ONCABG post-surgical outcome [20,21].

**Table 3:** Outcomes of postoperative renal failure-dialysis prevalence in the total study populations with on-pump and off-pump surgeries. OPCABG, off-pump coronary artery bypass graft; ONCABG, on-pump coronary artery bypass graft. Chi-squared test was utilised and a threshold for statistical significance of p < 0.05 was used [15,20,21].

	OPCABG		ONCABG		P-value
Study	Events	Total	Events	Total	
Shroyer et al. (2014) [21]	5	401	5	433	0.903
Magee et al. (2001) [15]	3	346	70	2545	0.0361
Song et al. (2024) [20]	5	187	4	187	1.000

In line with these findings, Song et al. conducted a large retrospective cohort study of 548 diabetic patients undergoing isolated CABG, with 352 treated via OPCABG and 196 via ONCABG [20]. Following 1:1 propensity score matching, 187 well-balanced patient pairs were analysed. The incidence of renal failure requiring dialysis within 30 days postoperatively was low in both groups, i.e., 2.7% in the OPCABG group and 2.1% in the ONCABG group, with no statistically significant difference between them (P = 1.000). Additionally, the study evaluated renal insufficiency as a secondary outcome, finding no significant differences between the groups. These results suggest that off-pump and on-pump CABG approaches yield comparable short-term renal outcomes, including both the need for dialysis and the development of renal insufficiency, in diabetic patients with three-vessel disease.

The outcome of RF requiring haemofiltration was assessed as a secondary outcome in the single-centre, cohort study conducted by Renner et al. [22]. The study sampled 857 diabetic patients, who were placed in different surgical groups, and their preoperative characteristics were measured. No statistically significant differences were identified between the groups in the preoperative parameters that could impact postoperative AKI, the exceptions being estimated glomerular filtration rate (eGFR), which was higher in the OPCABG group (P = 0.022), and gender, 6.4% more females in the ONCABG group (P = 0.037). Data on postoperative outcomes showed a statistically significant 7% increase in RF requiring haemofiltration associated with the ONCABG group (OR (95% CI) = 0.30 (0.16-0.58); P = 0.002). Propensity score adjustment of data did not alter the magnitude of this association (OR (95% CI) = 0.30 (0.14-0.64)) [22].

Outcome of Creatinine Clearance and Proteinuria as Measures of Renal Dysfunction

Disruption in creatinine kinetics is a common presentation of AKI and DN; thus, creatinine measurement can help distinguish the extent of postoperative renal damage in diabetic patients. SCr is measured using laboratory blood tests or by commercial assay kits. Plugging this value into the Cockcroft-Gault equation [23], or into the clearance equation, for more accuracy, calculates the CrCl rate. Normal CrCl range is 97 to 137 mL/min in men and 88 to 128 mL/min in women; anything outside this range can be indicative of renal dysfunction. Renal dysfunction in the form of AKI or DN can precipitate significant damage to the glomerulus, resulting in abnormally high protein excretion - proteinuria. The albumin excretion rate is a reliable measure of proteinuria, as a healthy glomerular filtration barrier prevents the passage of albumin. Microalbuminuria is characterised as an excretion rate of 30-300 mg/24 hours and is associated with renal dysfunction.

**Figure 6:**
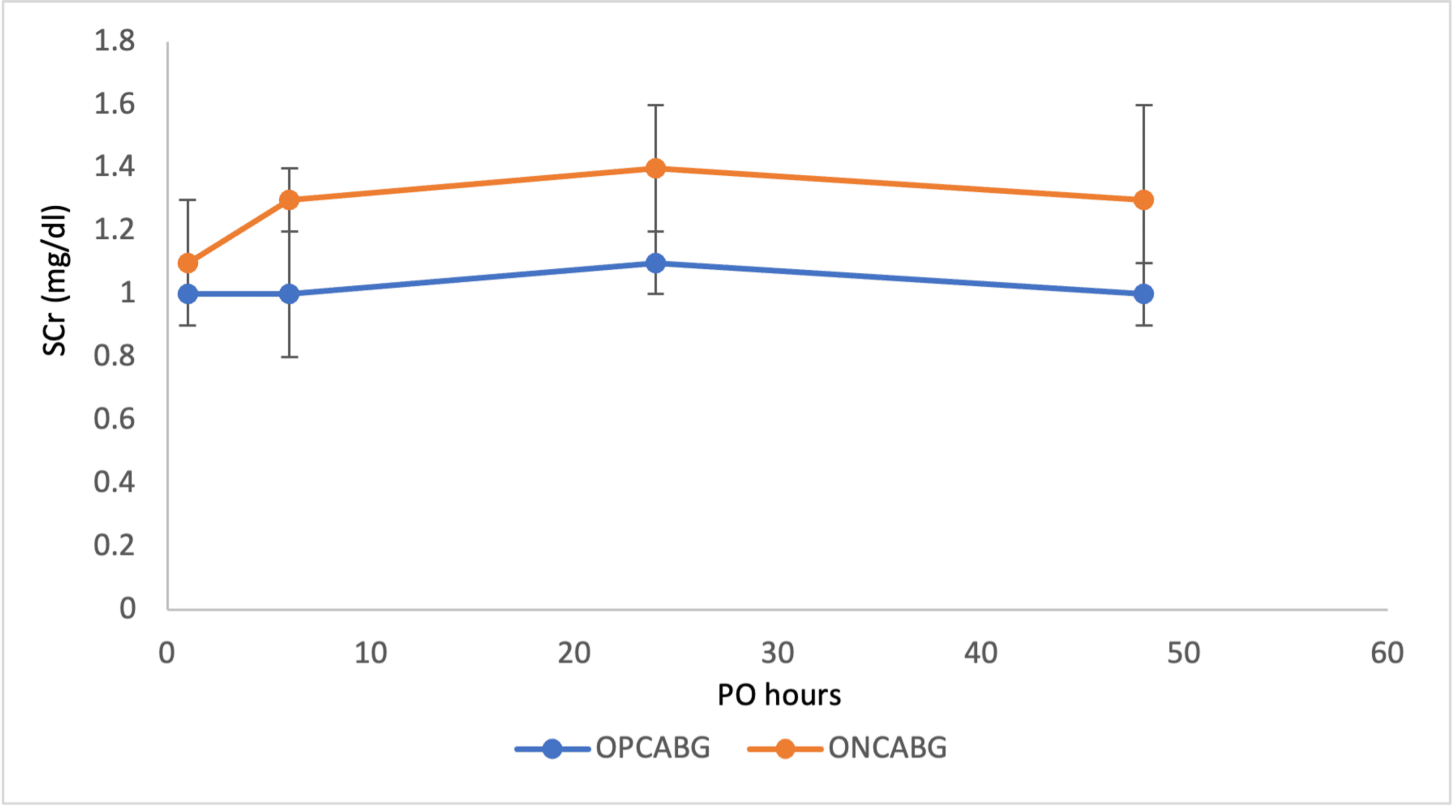
Dynamic changes in serum creatinine at six hours, 24 hours, and 48 hours post operation. The first dot plot at ‘PO = 1’ is the mean serum creatinine measurement of each treatment group at surgical admission, and the standard deviation is presented as error bars. SCr, serum creatinine; PO, postoperative; OPCABG, off-pump coronary artery bypass graft; ONCABG, on-pump coronary artery bypass graft. Data from Pramodh et al. [25].

**Figure 7:**
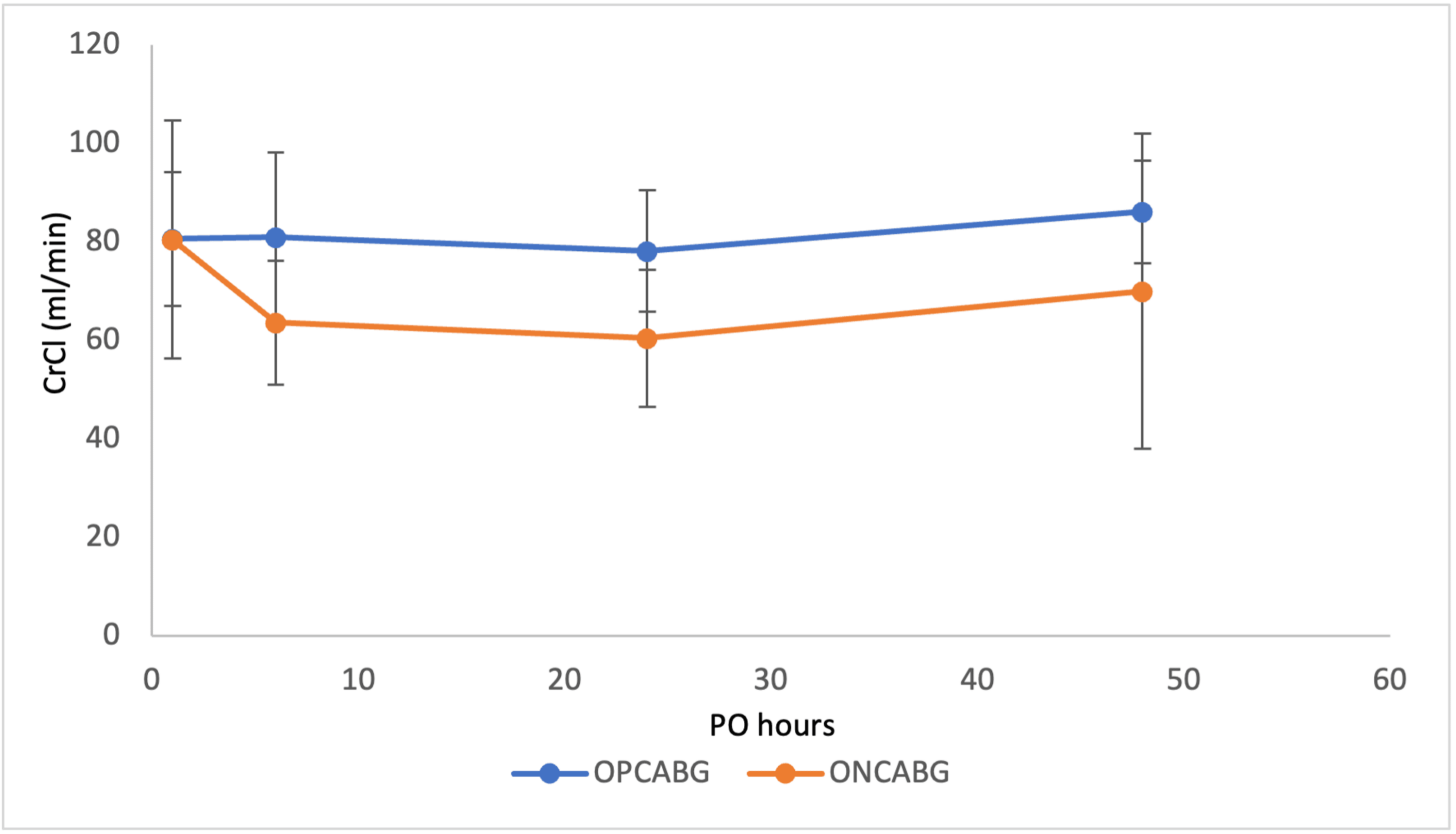
Dynamic changes in creatinine clearance at six hours, 24 hours, and 48 hours post operation. The first dot plot at ‘PO = 1’ is the mean CrCl measurement of each treatment group at surgical admission, and the standard deviation is presented as error bars. CrCl, creatinine clearance; PO, postoperative; OPCABG, off-pump coronary artery bypass graft; ONCABG, on-pump coronary artery bypass graft. Data from Pramodh et al. [25].

Single-centre randomised controlled trials conducted by Pramodh et al. and Zirak et al. enrolled 60 and 67 patients, respectively, into either ONCABG or OPCABG surgery [24,25]. Both studies quantified the effect of surgical strategy on the outcome of renal dysfunction by comparing postoperative CrCl of patients at six hours, 24 hours, and 48 hours with the baseline preoperative CrCl and against the standardised SCr limit of 1.3 mg/dl. The studies excluded patients with a history of RF with SCr > 1.3 mg/dl and included both diabetic and nondiabetic patients, with statistical comparisons made to highlight the impact of this comorbidity. In both studies, preoperative parameters between treatment groups were not compared within the diabetic subgroups, and no sample power analysis was conducted for the diabetic population. Therefore, these risks are major influences on renal outcome caused by these confounding variables. The postoperative data of Pramodh’s diabetic subgroup showed no statistically significant difference (p-values not published) in the evolution of mean SCr and CrCl at different time points in relation to surgery strategy. Despite the lack of statistical association, a trend can be identified in mean SCr and mean CrCl over time, as displayed in Figures 6, 7. Conversely, Zirak et al. considered an outcome of ±20% SCr fluctuation as renal dysfunction, with statistical analysis showing significant differences at 6th (P = 0.038) and 24th (P = 0.003) postoperative hours between the groups. This showed no significant correlation between diabetes and postoperative renal dysfunction between the groups (p-value not published) [24,25].

Quantification of creatinine outcome was also performed by Modine et al., alongside measurements of postoperative proteinuria and troponin Ic concentrations in OPCABG and ONCABG groups [26]. A total of 71 diabetic patients were enrolled, with 50.7% undergoing ONCABG. Measurements were taken every 24 hours, starting from surgical admission to the 5th postoperative day. Measurements and comparisons of all preoperative patient parameters that could precipitate AKI were conducted, and a statistically significant difference was only present in the median CrCl, 88 ml/min in ONCABG versus 68.8 ml/min in OPCABG (P = 0.0109). To account for the influence of this cofactor on the data, adjustments were applied using multivariate rank adjustments to normalise the difference in baseline CrCl. Adjusted postoperative outcomes showed no statistically significant difference in the median maximum CrCl level between the groups at 147.9 (IQR = 124.6; 169.9) ml/min in OPCABG and 130.1 (109.4; 143.1) ml/min in OPCABG. In contrast, statistically significantly higher maximum proteinuria and maximum troponin concentrations were obtained in the ONCABG group, with differences of 0.9 mg/24 hours (P <0.0001) and 1.0 μg/l (P = 0.0003), respectively. There was a trend towards higher maximum albuminuria in the ONCABG group (P = 0.06), with a significant difference in the six-day evolution of microalbuminuria, as the OPCABG group values returned faster to the baseline (P = 0.003) [26].

Assessment of the study quality and conduct

As indicated in Table 4, various limitations in study conduct were present in the studies included in this review. Lack of randomisation of patients was a limitation faced, most notably in the observational studies. Randomisation of a sufficient sample size would enable equal distribution of patient preoperative characteristics in the treatment groups. Consequently, this would yield accurate data that are representative of the effect of treatment on the outcome. Lack of randomisation makes a study vulnerable to selection bias and confounding, lowering the overall quality of the data. Some of the studies mentioned in this review used propensity score or multivariate model adjustments to minimise the effects of confounding factors. These are both statistically valid and robust methods to use for reducing the difference between the preoperative parameters of patient groups, utilising the alternatively stratified or matched data between groups to analyse treatment effect [27,28]. Renal function was considered the secondary outcome in two of the reviewed studies. This limitation reduces the accuracy of the overall outcome as the power analysis of a study focuses on the primary outcome, causing higher risks of type 1 and type 2 errors in secondary outcomes. Additionally, the measures of preoperative characteristics function to reduce confounding of the primary outcome, causing secondary outcomes to be less valid. Not publishing the p-values of analyses was a factor that affected the overall interpretation of study outcomes. A specific confidence interval is used as a threshold for assessing statistically significant differences in data. Any values outside of this threshold can potentially highlight insignificant trends in data that may be useful for the overall comparison. A common occurrence during OPCABG surgery is the need for conversion to CPB. This event can precipitate overlap in data; therefore, it must be measured and evaluated in the outcomes. Despite this, three of the studies failed to anticipate the treatment conversions or institute methods of dealing with their data. Four of the studies in this review failed to sample a large enough cohort of patients for the outcome of renal function. An insufficient sample size can increase the risk of type 2 errors and outliers. Performing the different CABG surgical strategies at similar time frames of the study would further avoid bias by ensuring that the same hospital staff carried out the procedures under the same routine. Having analysed all the study flaws, we can conclude that Shroyer and colleagues published the highest quality study included in this review.

**Table 4:** Limitations identified in different studies. Table made using resources [15-17,19,22,24-26].

Limitations	Studies
No randomisation	Magee et al. [15], Srinivasan et al. [16], Renner et al. [22], Benedetto et al. [19], Emmert et al. [17]
Renal function as a secondary outcome	Renner et al. [22]
No data on treatment conversions	Magee et al. [15], Zirak et al. [24]
Insufficient sample size	Srinivasan et al. [16], Pramodh et al. [25], Emmert et al. [17], Zirak et al. [24]
Statistical test values not published	Pramodh et al. [25], Zirak et al. [24], Modine et al. [26]
Different surgical strategies were conducted at different study periods	Srinivasan et al. [16], Emmert et al. [17]

Discussion

This is the first systematic review comparing the effect of OPCABG versus ONCABG on the renal function of diabetic patients. As this topic is relatively untouched by the scientific community, this review faced inevitable limitations put forward by the scarcity of relevant high-quality studies and the heterogeneity present between studies. The statistical heterogeneity was brought about due to the lack of control over confounding variables, resulting in the true treatment effect being different in different studies. In this context, the confounding variables are the preoperative parameters that could influence postoperative renal function. Their invalidating impact on the outcomes of each study is considered for the accurate interpretation of results in this section.

Confounding Factors

The independent risk factors of postoperative renal injury included in this review act through different mechanisms to induce susceptibility. Studies have shown advanced age (of >63 years old) to be an independent risk factor of post-CABG AKI [29-31]. This risk factor acts both directly and indirectly to precipitate renal injury. The direct impact is the progressive reduction in GFR by age that makes these individuals more susceptible to renal damage, mediated by the hypoperfusion during surgery. Intrinsically, females are categorised to be at significantly higher risk of post-CABG AKI due to their baseline lower eGFR at surgical admission. The correction of this difference removed the postoperative association between gender and AKI, highlighting gender as an independent risk factor [32]. Obesity and morbid obesity have also been identified as risk factors for postoperative AKI. F2-isoprostane is a product of the free radical-catalysed peroxidation of fatty acids. This biomarker of oxidative stress is elevated in obese patients, indicating an increased risk of AKI via damage to the glomerulus, reduced renal perfusion, and lower CrCl [29,33]. A higher number of diseased coronary vessels has been associated with postoperative AKI. This is theorised to be due to the longer operative times associated with the more complex nature of these lesions, therefore requiring longer periods of hypothermic circulatory arrest. Moreover, a higher percentage of chronic kidney disease is observed in patients with multivessel disease [34], leading us to the last preoperative parameter of this review, which is the presence of preoperative renal dysfunction. This risk factor is measured by comparing the preoperative incidence of RF, the rate of CrCl, or SCr concentration between the OPCABG and ONCABG groups.

Effects of Different CABG Surgical Strategies on the Outcomes of Renal Function in Diabetic Patients

Summary of the studies that focused on the outcome of RF do not indicate a clear benefit favouring a surgical strategy (as shown in Table 2 and Figure 5). The odds ratios of Emmert et al., Magee et al., and Srinivasan et al. [15-17] show stronger associations of data in favour of OPCABG surgery, with Srinivasan being the only study presenting a statistically significant difference in RF incidence. Individual considerations must be made when interpreting the overall outcome in these four studies. Magee et al.'s study (the study with the highest p-value) suffers from the influence of confounding factors in their data, showing more statistically significant differences in preoperative parameters between treatment groups and not including propensity score adjustments. With this allowance, more of an overall trend can be interpreted in favour of OPCABG.

The outcome of severe RF involved the filtered inclusion of patients with stage 3 of the Acute Kidney Injury Network (AKIN) classification of AKI [35]. This outcome was measured by the patient’s postoperative need for RRT, and the studies analysed produced quite different outcomes. Studies measuring postoperative incidence of RF-dialysis and haemofiltration by Magee et al. and Renner et al., respectively, showed statistically significant outcomes in favour of OPCABG [15,22]. Considering that only the latter study used an adjusted statistical model to counter confounding factors and that these findings are contradicted by the results of Shroyer et al. and Benedetto et al., no difference in severe renal outcome with respect to surgical strategy can be concluded. Supplementary to this conclusion, it is noteworthy to mention that the percentage incidence of RRT by Shroyer and Benedetto was in favour of ONCABG without statistical significance [19,21].

Lastly, three of the included studies quantified postoperative renal function by CrCl. Unfortunately, Pramodh et al. and Zirak et al. did not publish the p-values associated with their statistically insignificant comparisons, meaning that no insignificant trends could be deduced. They presented the lowest quality of data for the interpretation of renal outcome in diabetic subgroups, as they lacked the comparison of preoperative parameters and insufficiently powered their samples (high risk of a type II error). Overall, neither of these papers favoured a surgical option over another in the diabetic comparison; however, both did show statistically significant depression in CrCl at the 24th postoperative hour associated with ONCABG in the general comparison (including nondiabetics). Additionally, Pramodh's diabetic subgroup comparison showed a pattern of ONCABG associated with higher mean SCr and lower mean CrCl at all time points, insignificantly favouring OPCABG surgery [24,25]. Unlike these studies, Modine et al.'s measurements of postoperative CrCl considered the preoperative parameters and applied an adjusted data model to balance their impact [26]. Similarly, this paper did not show any significant difference between the surgical strategies in CrCl, but did associate OPCABG with better proteinuria and albuminuria outcomes. Fluctuations in proteinuria and albuminuria are a good representation of glomerular dysfunction. Higher postoperative proteinuria indicates the presence of DN, which elevates the risk of AKI.

ONCABG and Acute Kidney Injury

Renal impairment after cardiac surgery is multifactorial. Other than the preoperative parameters, intraoperative risk factors play a vital role in the renal outcome of patients. Despite their presence in both ONCABG and OPCABG surgeries, some of these risk factors are more deleterious with CPB. Frequently, complex operations, often involving treatment of repeat revascularisation in multivessel disease, rely on ONCABG. This increases the risk of AKI due to the associated longer surgical duration and increased inflammatory stimulation. Long durations of CPB are associated with higher risks of AKI and higher mortality rates [36], due to the amplified influence of systemic inflammation, haematocrit, hypoperfusion, and embolic congestion. The contact of blood with the foreign surfaces of the CPB circuit activates blood immune components and triggers an increase in IL-6, IL-10, IL-8, and tumour necrosis factor-alpha proinflammatory cytokines [37]. Inflammation is further aggravated by the ineffective filtration of microemboli during CPB, congesting the renal vasculature, therefore, causing I/R injury. Microemboli include atheroemboli from the aortic cross-clamping and cannulation, fat, cellular debris, or air embolisms. Non-pulsatile CPB flow alters the renal vasomotor tone by activating the sympathetic nervous system, inducing the renin-angiotensin system, and increasing the release of catecholamines, consequently, promoting hypoperfusion and hypoxia [38]. CPB influences the haematocrit via the damage inflicted on erythrocytes by circuit components, resulting in the increased circulation of plasma-free haemoglobin and labile iron. Plasma-free haemoglobin reduces renal NO production and can cause kidney injury by catalysing free radical production, and circulating labile iron promotes ROS production in the kidneys, further promoting inflammation and AKI [39]. Additionally, haemolysis drives complement activation, which contributes to the systemic inflammatory response and I/R injury of AKI [40]. Hypothermic perfusion temperatures of CPB have both positive and negative effects on renal function. The low temperatures reduce ischaemia by slowing metabolism, but the rapid shift from hypothermia to normothermia at surgical termination causes hypoperfusion of the superficial cortex, causing mitochondrial dysfunction and proapoptotic signalling [41]. These risk factors of AKI in CPB explain the trend in favour of OPCABG for reduced renal dysfunction in our results. The lack of benefit between severe RF requiring RRT and OPCABG might be indicative of the extent to which CPB can precipitate acute renal disease. The influence of diabetes and concomitant DN on the outcomes of surgical strategies is evaluated below.

Effect of Preoperative DN on the Outcomes of On-Pump Versus Off-Pump Surgery

DN is a condition that gradually develops in the diabetic kidney due to the persistent metabolic and haemodynamic factors. It is estimated that nearly half of patients with IDDM develop DN in their lifetime, and 29% to 38% of NIDDM patients present with an abnormal renal function associated with DN within a 15-year period [42,43]. This shows that most diabetic patients experience accumulation of microvascular insult that, at some stage, will precipitate their renal dysfunction. With this concept in mind, most of the studies analysed in this review have inadvertently considered the impact of DN on the outcomes within the overall diabetic population. To further isolate this impact, more valid studies need to be conducted, including studies that focus solely on the impact of ONCABG versus OPCABG on the renal function of diabetics with renal insufficiency. Additionally, characterisation of renal insufficiencies must include low eGFR, low CrCl, high SCr, or high albuminuria.

Renal status seems to have a profound impact on the outcomes of cardiac surgery. Studies have found preoperative renal functional changes to be associated with a substantial increase in risks of mortality and morbidity [44-46]. Slightest of changes that are within normal ranges of eGFR and SCr can significantly impact postoperative outcome to an extent where a 15 ml/min/1.73 m^2^ reduction in eGFR can precipitate a statistically significant increase in the risk of mortality [44]. Similarly, reductions in preoperative eGFR with adjustments for diabetes have been associated with significantly higher long-term mortality rates for patients undergoing cardiac surgery [47]. The comparison of surgical strategy on the outcomes of renal insufficient patients was made in a limited number of studies, but similar results were yielded. Sajja et al.'s analysis of diabetics with non-dialysis-dependent renal impairment identified a greater incidence of mortality and a 35.9% greater incidence of SCr elevation associated with ONCABG surgery [46]. Likewise, the study by Weerasinghe et al. found ONCABG, diabetes, SCr, and hypertension to be the strongest independent risk factors of postoperative renal dysfunction [45]. The benefit of off-pump surgery is pronounced for the survival benefit of these patients [46], but CPB seems to be more beneficial for the long-term survival outcomes of patients with severe preoperative RF. The observational study by Huang et al. showed that ONCABG provided a statistically significant 32% reduction in the long-term mortality rate of patients with concomitant diabetes and ESRD [48]. This relationship can be attributed to the more successful complete revascularisation present in ONCABG surgery as opposed to OPCABG [49].

In the diabetic renal insufficient population, besides the impact of CPB on renal impairment, the effect of DN on postoperative AKI must be considered. DN worsens the ischaemia vulnerability of renal tissue through endothelial dysfunction and aggravates inflammation. As previously mentioned, endothelial dysfunction can cause vasoconstriction through reduced NO production and increased endothelin-1 production and sensitivity [12]. Meanwhile, persistent hyperglycaemia drives apoptosis of the endothelium through vascular rarefaction mediated by nuclear factor kappa B (NF-kB) and c-Jun NH2-terminal kinase [13,50]. The hypoxia, caused by these factors, stimulates mitochondrial dysfunction that further exacerbates damage by ROS. These conditions predispose the kidneys to ischaemia and therefore AKI. DN kidneys have a higher baseline inflammatory status induced by the expression of various mediators that are also involved in the pathophysiology of AKI. DN kidneys have elevated chemokine and cytokine production by the proximal tubular cells, caused by hyperglycaemia, elevated levels of advanced glycation end products, and microalbuminuria that promote NF-kB signalling [51]. Furthermore, microalbuminuria induces ROS production, a major pathological factor of AKI, that, alongside NF-kB activation, drives the expression of IL-8 [52]. IL-8 is a proinflammatory stimulant for neutrophil activation that is directly involved in the pathogenesis of AKI following ONCABG surgery [53,54].

Review Limitations

Several limitations in this review, as previously mentioned, were caused by the low quality and scarcity of relevant studies. More lenient inclusion criteria could have increased the number of studies. Inclusion of retrospective cohort studies would introduce selection bias, information bias, and confounding.

Another limitation in this review was the limited consideration of confounding factors. As listed in Table 5, numerous preoperative, intraoperative, and postoperative factors that, if not controlled, could influence the postoperative renal function, albeit to different extents, if not properly controlled. Most of the included studies failed to control for the influence of all these factors between groups, and this review only focused on the most profound factors; therefore, increasing the risk of false associations. The accumulation of these factors precipitates statistical heterogeneity between studies that could have been quantified using the I^2^ test.

**Table 5:** Perioperative factors that influence postoperative acute kidney injury. Source [41].

Preoperative	Intraoperative	Postoperative
Chronic kidney disease	Complexity of surgery	Vasopressor exposure
Female gender	Emboli (cholesterol and other)	Inotrope exposure
Hypertension	Inotrope exposure	Diuretic exposure
Hyperlipidaemia	Hypovolemia	Blood transfusion
Advanced age		Anaemia
Obesity		Hypovolemia
Peripheral vascular disease		Venous congestion
Previous stroke		Cardiogenic shock
Smoker		

Separate comparisons were made to identify the impact of DN-mediated renal insufficiency and surgical strategy on the renal outcome of CABG surgery. Ideally, the studies analysed should have considered these factors simultaneously, limiting the analysis of these two variables to the same population and providing more valid data. Currently, there are no high-quality clinical trials that compare the outcome of renal function between surgical strategies in DN patients. Thus, future conduct of these studies is highly recommended.

Directions for Future Research

This review confirms the need for more randomised controlled trials on diabetic patients with or without renal insufficiency, comparing ONCABG versus OPCABG on their renal outcome. Such high-quality studies should be powered to ascertain AKI as a primary outcome using composite measures of GFR, SCr, and albuminuria. Also, they must ensure minimal statistical difference in the frequency of risk factors between treatment groups. Considering the link between DN and postoperative AKI, new techniques must be utilised to reduce the burden of preoperative renal insufficiencies. For example, the administration of exogenous albumin to patients suffering from hypoalbuminemia stemming from albuminuria has shown promising protective results against AKI in OPCABG patients [55]. DN and AKI are clinically associated via various shared inflammatory mechanisms. Premeditated administration of anti-inflammatory drugs such as simvastatin, at specific doses, could precipitate better renal outcomes of CABG by down-regulation of IL-8 production, preventing their clinical association. Other candidate nephroprotective drugs should be identified and evaluated by trials for clinical efficacy. Potential renal-protective alterations can improve the negative characteristics of on-pump surgery that precipitate AKI; these include optimisation of the rate of hypothermic to normothermic temperature shift, reduced inflammatory contact activation by miniaturised CPB and drug-coated circuits [56], and implementation of pulsatile pumps [57].

## Conclusions

Current data from clinical trials indicate that off-pump CABG offers comparable long-term outcomes to on-pump CABG while demonstrating potential advantages in reducing perioperative complications, particularly in high-risk patients. This review demonstrates a trend of higher quality data in favour of off-pump surgery in diabetic patients for the reduced incidence of RF and renal dysfunction. The same association cannot be made for postoperative incidence of severe RF requiring RRT. Highly concurrent comorbidity of diabetes with DN and the background pathophysiological associations of DN and AKI could imply a similar trend in favour of off-pump surgery. Further large-scale randomised trials are warranted to confirm these findings and guide individualised surgical decision-making.

## Appendices

**Table 6:** Review matrix appendix. CABG, coronary artery bypass grafting; CPB, cardiopulmonary bypass; OPCABG, off-pump coronary artery bypass graft; ONCABG, on-pump coronary artery bypass graft; OPCAB, off-pump coronary artery bypass graft; ONCAB, on-pump coronary artery bypass graft; PSM, propensity-score matching; STS, Society of Thoracic Surgeons; RF, renal failure; MI, myocardial infarction; AF, atrial fibrillation; BIMA, bilateral internal mammary artery; GFR, glomerular filtration rate; MACCE, major adverse cardiac and cerebrovascular events. Table made from resources [15-22,24-26].

Article	Author and date	Study design and purpose	Sample, sample size, and selection	Statistical analysis	Study findings relevant to the research question	Points to merit	Authors limitations	Reviewer’s limitations on the research question	Bias
Influence of diabetes on mortality and morbidity: off-pump coronary artery bypass grafting versus coronary artery bypass grafting with cardiopulmonary bypass	Magee et al. (2001) [15]	Retrospective review of prospective data gathered by the Society of Thoracic Surgeons (STS) of 9,965 patients who underwent CABG between 1995 and 1999. Aims: The impact of off-pump surgery as opposed to CPB on outcomes of diabetic patients relative to non-diabetic patients.	Sample of 2,891 diabetic patients: 346 off-pump; 2,545 on-pump. Selection: Selection of patients into CPB or off-pump groups was at the discretion of the operating surgeon and was dependent on the patients' calculated risk of complications due to CPB utilisation. Patient selection was contemporaneous.	Chi-squared test. Two-tailed Student’s T-test was used when involving an unknown scaling factor. Preoperative risk factors were calculated in correspondence to the preoperative patient characteristics. The STS algorithm was used to calculate the predicted risk of operative mortality in groups.	The diabetic off-pump group had statistically significantly lower incidence of postoperative RF-dialysis than the CPB group. In contrast to the observation in non-diabetic patients. The diabetic off-pump group had a lower percentage incidence of RF compared to CPB, but this outcome was not statistically significant.	Strong sample size with a good representation of routine practice.	Lack of randomisation of patients selected to undergo either off-pump or on-pump surgeries. Creating selection bias.	Mean age and percentage of female participants were both higher in the off-pump diabetic group compared to the CPB diabetic group. Confounding factors increase the risk of postoperative renal failure. More single CABG grafts in the off-pump group than the CPB group. More multivessel disease is seen in patients undergoing CPB surgery.	Selection bias
Renal function following CABG: On-pump vs off-pump	Pramodh et al. (2003) [25]	Prospective randomised controlled trial. Aim: Assessment of renal function in patients undergoing on-pump and off-pump surgeries. Secondary identification of the impact of hypertension, diabetes and ejection fraction <40% on the post-surgical development of renal dysfunction in the groups.	A sample of 60 patients, with 30 undergoing on-pump and 30 undergoing off-pump surgeries. Randomisation was strictly adhered to as no cross-over from off-pump to on-pump occurred.	Student’s t-test to enable comparison of the two independent groups. T-test with modified degrees of freedom was used when appropriate. The Mann-Whitney U test was used for comparisons. Chi-squared test/Fisher’s exact test, and Pearson’s correlation coefficient. Four-way repeated measures multivariate analysis of variance.	Creatine and creatine clearance of patients with diabetes in the two groups were compared. Although the means at 6 hours, 24 hours, and 48 hours show a trend in favour of off-pump, there was no statistical significance when compared using Fisher’s exact test.	Patients randomised. No treatment crossovers/conversions.	No limitations stated.	P-values not published on the difference in post-surgical creatinine clearance in diabetic patients undergoing either on-pump or off-pump surgery. Risk of type 2 error in identifying any existing difference in diabetic renal function of patients undergoing on-pump or off-pump surgeries. No sample power analysis. No pre-operation demography of the diabetic subgroup. No pre-operative consideration of differences in baseline creatinine clearance between groups.	Risk of reporting bias
Coronary revascularization in diabetic patients: off-pump versus on-pump surgery	Renner et al. (2013) [22]	Single-centre, cohort study isolating 857 adult diabetic patients from a pool of 3,34 patients who underwent open-heart surgery. Aims: The study investigated if off-pump CABG has an effect on 30-mortality and mid-term mortality of diabetic patients. Secondly, looking at the impact of off-pump surgery on postoperative complications.	A sample of 857 diabetic patients, with 355 undergoing off-pump surgery and 502 undergoing on-pump surgery. Selection: The choice of on-pump or off-pump group selection was based on patients' risks and benefits.	Logistic regression analysis. Cox regression analysis. Fisher’s exact test (categorical variables) and Mann-Whitney U test (continuous variables). Analysis of postoperative haemofiltration requirement between the groups was done by utilising analysis of covariance (ANCOVA), with propensity score and EuroSCORE as covariates.	Postoperative requirement for haemofiltration as a secondary outcome. Statistically significantly lower percentage of patients received haemofiltration in the off-pump group.	Propensity score – adjusted analysis used to control for selection bias. Intention-to-treat methodology.	No randomisation was in place. Single-centre study may not be a good representation of overall surgical practice.	The estimated preoperative GFR was higher in the off-pump group. No statistics on the preoperative albuminuria. Significant gender difference between groups. Sample power analysis not mentioned, risk of type 2 error. Risk of type 1 error due to a high number of statistical tests used to derive p-values.	Risk of selection bias
On-pump versus off-pump coronary artery bypass grafting in diabetic patients: a propensity score analysis	Srinivasan et al. (2004) [16]	Data collected prospectively in the single-centre study of patients undergoing CABG. Aim: Evaluate the outcome of diabetic patients undergoing off-pump with on-pump CABG.	951 propensity-matched diabetic patients, with 186 undergoing off-pump surgery and 765 undergoing on-pump surgery. Selection: The decision of treatment used depended on the patient’s clinical presentation and the surgeon’s preference.	Odds ratio analysis and logistic regressions. P-values derived using Wilcoxon rank sum tests and Chi-squared tests. The propensity score was constructed using preoperative variables of patients.	Propensity-adjusted and crude analysis of postoperative renal failure (%). Crude analysis showed a trend in favour of off-pump surgery, whilst propensity matching showed a significant reduction in the percentage of renal failure associated with off-pump surgery. After the removal of 43 patients who had preoperative renal failure from the analysis, the results still showed a trend in favour of off-pump surgery.	Intention-to-treat analysis was implemented to account for any crossovers. Propensity matched to avoid selection bias. Data on preoperative renal dysfunction showed no significant difference between groups.	No randomisation observational study. Some off-pump surgeries took place during a teaching period of the surgeons. 5-year study with most on-pump surgeries taking place in the earlier period of the study, whereas the off-pump surgeries took place in the later stages of the study.	Significantly more patients with three-vessel disease underwent on-pump surgery. No data on long-term renal outcome. No data on preoperative GFR. More preoperative obesity in off-pump patients.	Selection bias
Off-pump versus on-pump coronary artery bypass surgery in patients with actively treated diabetes and multivessel coronary disease	Bendetto et al. (2016) [19]	Observational study, retrospective analysis, single-centred study of all isolated first-time CABG procedures at the Bristol Heart Institute. Aims: Analysis of the short-term outcomes (including need for renal replacement therapy) and long-term survival in diabetic patients undergoing off-pump vs. on-pump CABG surgery.	A sample of 2450 diabetic patients, of which 1253 underwent off-pump surgery and 1197 underwent on-pump surgery. Selection: Patients underwent different modes of CABG based on the surgeon’s preference.	Multivariable logistic regression for propensity matching. Matched patients were checked for balanced covariate distribution by analysis of standardised mean difference. Time-segment Cox regression models. Pearson correlation coefficient and variance inflation factors.	No significant difference between on-pump and off-pump groups in postoperative percentage need for renal replacement therapy.	A propensity score was generated for each patient from a multivariable logistic regression model of pre-surgery covariates to reduce the impact of confounding factors.	No follow-up data available for long-term outcomes.	Non-specific design of the diabetic population limits the present analysis, adding to confounding factors.	Selection bias.
Is off-pump superior to conventional coronary artery bypass grafting in diabetic patients with multivessel disease?	Emmert et al. (2011) [17]	Single-centre cohort study, retrospective analysis of prospective data. Aim: Assessment of safety, feasibility, and completeness of revascularisation for diabetic patients undergoing off-pump vs. on-pump CABG.	Sample of 1015 diabetic patients with multivessel disease, of which 540 underwent off-pump and 475 underwent on-pump CABG. Selection: Based on the patient's clinical presentation and the surgeon’s preference.	Mean ± standard deviation and Mann-Whitney test in comparison of continuous data. Percentages, chi-squared, or Fisher’s exact test in comparison of categorical data. Logistic regression was used to compute odds regressions.	A composite endpoint measured was renal failure. Crude patient data outcomes showed no significant difference in renal dysfunction between the groups, but showed a trend in favour of the off-pump group. Propensity-scored matched data showed no significant difference in renal dysfunction between the groups. Showing less of a trend in favour of off-pump surgery.	The propensity score computed using 52 preoperative covariates, used to counter confounding factors. Intention-to-treat methodology.	Identification of surgeon and treatment differences is difficult due to the retrospective nature of the study. Insufficient patient cohort size. 6-year study period, where a major part of off-pump surgeries was from the later part of the study, as opposed to on-pump surgeries.	Off-pump patients had significantly lower EuroScore and less multivessel disease. Propensity score analysis variables not disclosed.	Selection bias
Comparing off-pump and on-pump clinical outcomes and costs for diabetic cardiac surgery patients	Shroyer et al. (2014) [21]	Analysis of a multi-centre, prospective, randomised controlled trial. Aim: To compare the primary and secondary study endpoints in diabetic patients undergoing either on-pump or off-pump surgery.	Sample of 835 actively diabetic patients, of which 402 underwent off-pump and 433 underwent on-pump CABG. Selection: Patients were randomised to different surgery types.	Continuous bivariate analysis performed by a t-test or Wilcoxon rank sum test. Categorical variables were tested with the chi-squared test or Fisher’s exact test. In addition to the intention-to-treat analysis, a secondary analysis was conducted, excluding the treatment conversions.	Primary short-term composite outcome of renal failure requiring dialysis – no differences between the treatment groups.	Intention-to-treat methodology. P-value adjusted to account for the multiple comparisons – p<0.01 as significant.	The unique risk profile of veterans cannot be generalised to the entire population.	A higher percentage of off-pump patients received fewer grafts than originally estimated. No measure of diabetes, degree of disease severity. No data on the number of patients with type 1 or type 2 diabetes.	Considered as low risk of bias by the Cochrane team.
Renal function following On-pump versus Off-pump CABG	Zirak et al. (2014) [24]	Prospective randomised clinical trials. Aim: Compare the postoperative renal function outcome of on-pump and off-pump strategies.	A sample of 67 patients, of which 34 underwent on-pump and 33 underwent off-pump surgery. Selection: Patients were randomised to each treatment group.	Chi-squared and Fisher’s exact test. Unpaired Student's t-test.	Either a 20% increase or decrease in serum creatinine is considered renal dysfunction. Significant difference in renal dysfunction between the two groups at 6 and 24 hours. No significant difference at 48 hours. When considering diabetes, the outcome had no impact on renal dysfunction.	Statistical analysis was performed using SPSS 15.0 software. The chi-squared test or the Fisher exact test was used for the categorical data, and unpaired Student's t-tests were employed to compare the continuous variables. A p-value<0.05 was considered statistically significant.	Not specified.	The sample was not powered to identify the difference caused by diabetes. No data on the impact of diabetes on the outcomes. No mention of power analysis in the diabetic subgroup.	Low risk of bias
A prospective randomized study to evaluate the renal impact of surgical revascularization strategy in diabetic patients	Modine et al. (2010) [26]	Single-centre, prospective randomised control trial. Aim: Evaluate the outcome of adverse renal outcomes in patients between on-pump and off-pump CABG.	A sample of 71 diabetic patients, of which 36 underwent on-pump and 35 underwent off-pump CABG. Selection: Patients were allocated to the treatment groups using balanced units of randomisation, the day before surgery. No crossover was experienced.	Quantitative variables were compared by the chi-squared test or Fisher’s exact test. Continuous parameters were compared by a Student t-test or Mann-Whitney U-test. Creatinine clearance differed at baseline between the two groups; thus, the analysis of covariance after rank transformations was used to create an adjusted model.	Renal function was assessed in patients by 24-h urinary collections starting from surgical admission (baseline measurement) to the 5^th ^postoperative day. Creatinine clearance calculated using plasmatic and urine creatinine levels. Urine albumin excretion rate calculated. Urine and plasma osmolality were calculated as a measure of tubular function. Glycemia, proteinuria, and serum troponin Ic concentrations were tested. Maximum albuminuria in the 5-day period between the groups showed a trend in favour of off-pump surgery, with the value reaching baseline faster. Maximum proteinuria showed a statistically significant lower outcome in off-pump surgery, and maximum troponin level showed a statistically significant lower outcome in off-pump surgery. Creatinine clearance was higher every day with on-pump surgery.	Power calculation conducted for the sample size.	Not specified.	Troponin IC is not very specific for renal dysfunction. The percentage of obesity is higher in the off-pump group. Preoperative patient characteristics highlighted as “NS” – not significant, does not enable identification of a possible ‘trend of difference’ with reference to the P-value.	Low risk of bias
Off-pump vs. on-pump bypass surgery grafting in diabetic patients with three-vessel disease: a propensity score matching study	Song et al. (2024) [20]	A retrospective cohort study aimed to compare early outcomes of OPCAB vs. ONCAB in diabetic patients with three-vessel coronary artery disease.	A total of 548 diabetic patients (OPCAB = 352, ONCAB = 196); after 1:1 nearest neighbour propensity matching, 187 matched pairs were included.	Student’s t-test, Mann–Whitney U-test, χ² test, Fisher's exact test, paired t-test, Wilcoxon test; PSM to adjust baseline.	OPCAB showed significantly reduced stroke (1.1% vs. 4.8%), respiratory infection (2.1% vs. 7.0%), and ventilator time; no significant difference in death, MI, or renal failure requiring dialysis.	Balanced matching, experienced surgeons, multiple outcomes assessed, detailed perioperative data.	Single-centre, retrospective, no long-term follow-up, excludes unmatched cases; diabetic control strategies not analysed.	Cannot assess long-term graft patency or MACCE; generalizability limited to experienced surgical centres.	Selection bias pre-matching; residual confounding; limited external validity
On-pump or off-pump impact of diabetic patient undergoing coronary artery bypass grafting 5-year clinical outcomes	Xu et al. (2024) [18]	Retrospective, single-centre study to compare in-hospital, 2-year, and 5-year outcomes of OPCAB vs. ONCAB in diabetic patients.	1269 diabetic patients undergoing primary CABG (OPCAB = 614, ONCAB = 655); 504 matched pairs after PSM.	Logistic regression, Cox proportional hazards, Kaplan-Meier survival; PSM used for adjusted comparisons.	Off-pump was associated with significantly lower 5-year mortality (4.2% vs. 7.1%, p = 0.041), lower stroke and AF rates; no significant differences in MI or renal failure.	Large sample, robust long-term outcome analysis, PSM confirmed consistency, and real-world relevance.	Retrospective, observational, surgeon selection not randomised; BIMA not evaluated; limited to single internal mammary artery use.	Does not stratify by diabetes severity or glycaemic control; limited data on completeness of revascularisation; generalisability.	Physician preference may affect treatment allocation; potential residual confounding despite PSM.
